# Optimizing the lignin nanoparticles from different pulping by-products in developing cotton-based nanocrystalline cellulose for UV‑light blocking

**DOI:** 10.1038/s41598-025-11489-7

**Published:** 2025-07-25

**Authors:** Altaf H. Basta, Vivian F. Lotfy

**Affiliations:** Cellulose & Paper Dept, National Research Centre, El Buhouth St., Dokki, Giza, 12622 Egypt

**Keywords:** Green disposing rice straw-pulping black liquors, Lignin nanoparticles, Nanocrystalline cellulose suspension, High performance UV shielding nanocomposites, Nanoscience and technology, Other nanotechnology, Nanometrology

## Abstract

The development of sustainable and non-toxic ultraviolet (UV) shielding materials is essential to address the limitations of conventional inorganic agents, which often suffer from biotoxicity and limited spectral coverage. In this study, lignin nanoparticles (LNPs) were extracted from rice straw-derived black liquors, a by-product of various pulping processes, using acid precipitation followed by solvent exchange with tetrahydrofuran (THF) and ethylene glycol (EG). These LNPs were incorporated into nanocrystalline cellulose (CNC) matrices to fabricate bio-based UV-shielding nanocomposites. The nanoparticles and their corresponding composites were characterized using transmission electron microscopy (TEM), attenuated total reflectance–Fourier transform infrared spectroscopy (ATR-FTIR), X-ray diffraction (XRD), atomic force microscopy (AFM), and polarized optical microscopy (POM). The nanocomposites demonstrated enhanced UV-blocking efficiency with increasing LNP loading (1–5 wt%), achieving up to 99.9% shielding in the UVC (200–280 nm) and UVB (280–320 nm) regions. Notably, CNC–LNP composites prepared using LNPs from Kraft pulping [Via tetrahydrofuran (THF); LNPT7)]and KOH/NH₄OH pulping [via Ethylene glycol (EG); LNPE6] exhibited nearly complete UVA protection. LNPE6 was characterized by semi-spherical particles with an intermediate average size of 23.8 ± 7.9 nm, whereas LNPT7 exhibited fully spherical particles with a significantly larger average size of 524.6 ± 233.6 nm. These findings highlight the potential of valorizing industrial lignin waste for the development of environmentally friendly, high-performance UV-protective materials for applications in packaging, personal care, and optical devices.

## Introduction

Sustainable development depends on increasing domestic energy efficiency since residential energy use accounts for a sizable portion of global energy consumption^1^. The utilization of renewable resources as an environmentally friendly substitute is growing as sustainability becomes a top priority for all industrial sectors^2^. Lignin, which is the second most prevalent naturally occurring aromatic biopolymer on Earth, is accepted by the plants for their stiffness, mechanical support, disease resistance, and ability to transport water and nutrients. It is a useful byproduct of the cellulose, paper, and biofuel industries because of its distinct qualities, including its chemical structure, hydrophobic qualities, and phenylpropane skeleton^3^. Our published research has demonstrated the potential of better environmental performance by introducing biowastes and their extractives (cellulose, lignin and nanosize forms) in manufacturing valuable products, such as carbon bio-adsorbent, bioactive compounds, hydrogel for sustain release of active compound, building units (cement mortars), rubber composites, optical materials and flocculants, and dispersants^4–12^. Furthermore, cellulose derivatives can be incorporated into composite materials for a wide range of applications. For example, composites of carboxymethyl cellulose (CMC) with ZSM-5 zeolite have demonstrated superior catalytic activity in acetalization reactions^13^. The same CMC–ZSM-5 composite, when combined with polyvinyl alcohol (PVA) and/or sodium alginate, has also been employed for efficient dye adsorption from aqueous solutions^14,15^. Additionally, recent advancements in cellulose- and alginate-based hydrogels have shown promising potential in water and wastewater treatment applications^16^.

Lignin has become a popular low-cost byproduct of the pulp and paper industry, which produces more than 30 million tons annually. By 2015, the world produced around 100 million tons of paper annually; by 2025, that amount is predicted to rise to $913.1 million^17^. The development of lignin nanoforms and the rise in publications utilizing lignin in agriculture have led to development of a wide variety of applications for lignin. A key strategy for preventing lignin aggregation and achieving lignin stability is the creation of lignin nanoparticles (LNP)^18^. By rupturing hydrogen bonds inside the lignin molecule, a green solvent called deep eutectic solvent may be utilized to break down C–C and ether bonds on the side chain of the benzene ring. A variety of techniques can be used to create LNPs, such as co-assembly or assisted formation with supercritical CO_2_ treatment^19^, homogenization by mechanical or ultrasonication^20^, electrospinning^21^, ice segregation^22^, solvent exchange^23^, cross-linking/polymerization, aerosol processing, and pH shifting^24^.

Commonly utilized UV shielding agents are inorganic UV shielding agents, e.g., alumina, silicon dioxide, titanium dioxide, and zinc oxide that mainly reflect and refract UV with differing indices of refraction than the matrix material^25,26^. These materials are frequently biotoxic, unstable and have limited absorption range and tendency to aggregate making them unsuitable for use as efficient UV adsorbent. As a biologically based UV shielding agent, lignin (an aromatic polymer developed of three phenylpropane units), offers a wide range of potential uses. However, its high value-added uses as a UV shielding material are limited by its heterogeneity and complexity. The last technique provided LNPs have its functional groups, aggregation shape, and surface polarity efficiently adjusted to enhance photo thermal conversion and UV shielding capabilities^27^.

In this study, lignin nanoparticles (LNPs) were extracted via acidification from various black liquors (BLs) obtained from different pulping methods of rice straw biomass. Subsequently, LNPs were synthesized through a solvent exchange technique using ethylene glycol (EG) and tetrahydrofuran (THF) in aqueous media. These LNPs were then incorporated with cellulose nanocrystals (CNCs) to form hybrid nanocomposites for ultraviolet (UV) shielding applications. The work aims to elucidate the influence of the black liquor source and LNP particle size distribution on the UV-blocking efficiency of the resulting nanocomposites. Comprehensive characterization techniques, including UV-Vis. spectroscopy, X-ray diffraction (XRD), attenuated total reflectance–Fourier transform infrared spectroscopy (ATR-FTIR), atomic force microscopy (AFM), and polarized optical microscopy (POM), were employed to investigate the structural interactions between LNPs and CNCs.

## Material and experimental techniques

### Materials

Cotton linter was provided from Factory 18 Military, Abu Zaabal, Egypt. Sulfuric acid was supplied from Merk. BL was produced by pulping rice straw with NaOH, NaOH with additives (Anthraquinone [AQ] and/or Sodium Borohydride [BH]), KOH-NH_4_OH, NaSO_3_-Na_2_CO_3_, and NaSO_3_ pulping agents in autoclaves with a liquid ratio of 6:1 at 120 °C^5^. The elemental analysis of the dried BLs is shown in Table 1 using Profilenc Technologies ECS 8020 CHNS-O (Italy). Additionally, the pulped RS’s chemical examination showed that silica and lignin components were also detected. Tetrahydrofurane (THF) and ethylene glycol (EG) were acquired from Nice-chemicals and S.D. fine chemicals.

**Table 1:** Pulping condition, CHNS-O elemental analysis in different black liquors and the removed lignin and silica from RS in BLs.

Conditions of RS-pulping	Code	Elemental analyses							Removed lignin and silica in BLs		
		C, %	H, %	N, %	S, %	O, %	H/C	O/C	Lignin removal, %	Silica removal, %	CNSs yield, %
NaOH (18.6% Na_2_O)	BL1	66.69	4.05	---	---	19.7	0.061	0.295	34.86 ± 1.74	57.96 ± 2.32	55.58 ± 1.95
NaOH (18.6% Na_2_O)-1%AQ	BL2	65.33	4.11	---	---	15.2	0.063	0.233	26.96 ± 1.45	48.41 ± 1.94	63.57 ± 2.23
NaOH (18.6% Na_2_O)-3%BH	BL3	63.75	4.08	---	---	16.3	0.064	0.256	13.32 ± 0.67	41.6 ± 1.66	54.39 ± 1.90
NaOH (18.6% Na_2_O)-1%AQ-3%BH	BL4	66.31	4.44	---	---	18.5	0.067	0.279	27.66 ± 1.38	48.84 ± 1.95	65.05 ± 2.28
M-NaOH (18.6% Na_2_O)-1%AQ-3%BH	BL5	65.27	4.37	---	---	17.3	0.067	0.265	39.42 ± 1.97	58.09 ± 2.32	64.95 ± 2.63
KOH/NH_4_OH (1:5)	BL6	57.85	5.11	12.51	---	20.32	0.088	0.351	17.81 ± 0.89	15.42 ± 0.62	38.95 ± 1.36
NaOH-Na_2_S (10% active alkali and 25% sulfidity)	BL7	58.9	5.1	---	4.95	21.2	0.087	0.360	73.11 ± 3.66	64.82 ± 2.59	17.16 ± 0.60
Neutral sulfite (Na_2_SO_3_/Na_2_CO_3_ [4:1])	BL8	60.83	4.89	---	5.5	16.7	0.080	0.275	10.83 ± 0.55	14.6 ± 0.58	17.15 ± 0.56
acidic sulfite (Na_2_SO_3_) (18.6% Na_2_O)	BL9	59.88	4.91	---	5.06	20.13	0.082	0.336	18.27 ± 0.91	13.78 ± 0.55	19.26 ± 0.68

### Experimental

#### Cellulose nanocrystals preparation and characterization

Cellulose nanocrystals (CNCs) were synthesized by hydrolyzing the cotton linter with sulfuric acid. In brief, 200 ml of 64% sulfuric acid was combined with 10 g of cotton linter, portion by portion, in an ice bath. Upon complete addition, the slurry was heated to 60 °C for 30 min while being constantly stirred. Centrifugation was used to wash the resultant product multiple times. After acid removal, the sample was dialyzed against distilled water until it reached a neutral pH. The prepared CNCs were subjected for further analysis included TEM, XRD, and FTIR utilizing a JEOL (JEM-1230 FX, Japan) microscope, a Bruker D8 advance (40 mA and 40 kV), and a VERTEX 80v-FTIR spectrophotometer, all made in Germany. The elemental composition of cellulose nanocrystals was explored using X-ray photoelectron spectroscopy (XPS; Thermo Scientific TM K-Alpha TM) with Al-K micro-fused monochromator operated at energy up to 4KeV.

#### Lignin nanoparticles extraction (LNPs)

According to the reference^28^, the concentrated black liquid was acidified to pH 4 using 20% v/v of phosphoric acid in order to precipitate the black liquor lignin. By heating the solution, the lignin that precipitated was gathered and dried. The method outlined in references^29,30^, was used for producing the lignin nanoparticle. Separately, EG or THF were used as a polar solvent to dissolve the dried extracted lignin at a concentration of 10 mg mL^− 1^ with stirring. After each solution was filtered, it was placed on a cellulose membrane for dialysis tubing from Sigma-Aldrich (D9527, which has an average flat diameter of 43 mm). It was then submerged in distilled water, which was refreshed on regular intervals. For at least twenty-four hours, the dialysis procedure produced lignin nanoparticles. The final suspension has been reserved for a further examination.

#### Characterization of LNPs

##### Transmission electron microscope (TEM)

The TEM pictures of LNPs were captured using a JEOL (JEM-1230 FX, Japan) microscope running at 200 kV. On 200 mesh copper-coated carbon grids, a few drops of diluted aqueous sonicated solutions were placed and left to dry for further examination. Particle dimension distribution (PDD) was determined indirectly by utilizing ImageJ software to process the TEM pictures. In order to determine the particle dimension distributions, several lengths and widths can be used to characterize the spherical or semi-spherical particles of LNPs.

##### ATR-FTIR spectral analysis

FTIR analysis was performed on the produced BL and LNPs using a VERTEX 80v-FTIR spectrophotometer made in Germany. To assign the function groups of the produced sample, the spectrum was obtained in the 400–4000 cm^− 1^ range.

#### Preparation CNC-nanocomposites

A stable a suspension with a concentration of 0.5 wt % of CNCs was prepared by sonication for 30 min after diluting CNCs in a suitable volume of deionized water. In the meanwhile, lignin nanoparticle homogeneous solutions in varying weight percentages (1, 3, and 5wt %) were produced independently and mixed with CNCs by sonicating them for 30 min. CNCs nanocomposites containing 5 weight% of other LNPs were prepared and then submitted to additional analysis.

#### Characterization of CNC-nanocomposites

##### Optical property (UV blocking)

To assess the UV shielding, the optical performance of nanocomposite suspensions was measured at 200–800 nm using a UV-Visible Single Beam spectrophotometer (UV1720, USA). For additional analysis, the leftover materials were dried and formed into films. The following formulas were used to determine the CNC-nanocomposites suspension’s UV blocking efficiency based on the transmittance data^31^.

$$\:\varvec{U}\varvec{V}-\varvec{b}\varvec{l}\varvec{o}\varvec{c}\varvec{k}\varvec{i}\varvec{n}\varvec{g},\:\varvec{\%}=100-\:\frac{\underset{\varvec{a}}{\overset{\varvec{b}}{\int\:}}\varvec{T}\left(\varvec{\lambda\:}\right)\varvec{d}\varvec{\lambda\:}}{\underset{\varvec{a}}{\overset{\varvec{b}}{\int\:}}\varvec{d}\varvec{\lambda\:}}$$

##### ATR-FTIR spectral analysis

FTIR analysis of CNCs-nanocomposites was performed using a VERTEX 80v-FTIR spectrophotometer, manufactured in Germany. Spectral data was gathered between 400 and 4000 cm^− 1^ in order to assign the function groups to the produced sample.

##### XRD test

The X-ray diffractometer Bruker D8 advance (40 mA and 40 kV), Germany, was used to obtain the XRD pattern of CNCs-nanocomposites utilizing Cu-Kα radiation. On a 2θ scale, the scanning speeds used to record the diffraction patterns ranged from 5° to 80°. The crystallinity index (CrI) was determined using the peak height method described by Segal et al.^32^, according to the following equation:

$$\:\varvec{C}\varvec{r}.\varvec{I}.=\frac{{\varvec{I}}_{002}-{\varvec{I}}_{\varvec{A}\varvec{M}}}{{\varvec{I}}_{002}}\times\:100$$

##### Atomic force microscopy (AFM)

Using AFM (Agilent 5600LS AFM system, USA), the topography, morphology, and roughness of the CNC and CNCs-nanocomposites films were examined. Under normal circumstances, the microscope was used and photographed in tapping mode.

##### Polarizing optical microscope (POM)

The CNCs-nanocomposite samples were examined using an Olympus Polarizing Microscope model BX-53-P with a lamp housing U-LHLEDC100, a rotatable analyzer (U-AN360P), a condenser (U-POC-2), a Bertrand lens (UPLFLN-P SERIES) with magnifications ranging from 4X to 100X, and Olympus cell sensors.

## Results and discussion

There are some noticeable changes in the bands shift and intensity of several bands following the extraction of lignin nanoparticles from the BLs. After the synthesis of nano-lignin, a new shoulder is observed at 1725 cm^− 1^ that is related to C = O of LNPs. Beside a reduction in the OH stretching frequency was observed; this might be due to the loss of β-O-4 links as well as a decrease in hydrogen bonds within the lignin molecules. Additionally, the increased resonance of the oxygen function groups of the LNPs causes an increase in the band intensity of the OH stretching (at ~ 3200–3400 cm^− 1^) and C-O bending (at ~ 1034–1080 cm^− 1^). On the opposite side the band at 1454 cm^− 1^ (C = C) showed reduction in the band intensity due to the loss bond in the lignin structure.

### Elemental analysis of black liquors (BLs)

Table 1 displays the elemental analysis of dried black liquors as lignin sources. The lignin and other low-molecular components present in black liquors are generally correlated with these elements. The ranges of BLs’ carbon, oxygen, and hydrogen contents are 57.85–66.69%, 15.20–21.20%, and 4.05–5.11%, respectively. The pulping reagents BL1–BL5, which are associated with NaOH or NaOH additives, have the highest carbon concentration (more than 60.00%). Due to their highest lignin removal rates (34.86 and 39.42%, respectively) from RS pulping, the BL1 and BL5 produced high carbon contents of 66.69% and 65.27. Regarding Kraft-BL (BL7), which has the maximum removal of lignin and silica (73.11% and 64.82%, respectively), resulting in distributed moderated carbon at 58.90% and the highest oxygen content at 21.20%. While nitrogen-containing BL is created by using KOH/NH_4_OH pulping reagent (BL6; 12.51% of N-content), sulfur-containing BLs (4.95–5.50%) is characterized by specific BLs that were produced by pulping by sulfur containing reagents (BL7–BL9) as a result of Kraft, neutral sulfite, and sulfite pulping. Based on the aforementioned information, BLs with a moderate to high lignin removal (10.83–73.11%) are useful for preparation of LNPs. The addition of BH and/or AQ to the NaOH pulping reagent has almost no effect on the hydrogen to carbon element (H/C) ratio of BL (6.10–6.70 × 10^− 2^). However, this value increased when other BLs (BL6-BL9) were used, the highest value for Kraft-BL (BL7; 8.7 × 10^− 2^) shows the lignin structure in black liquor, where the largest delignification percentage (73.11%) occurred. BL6, BL7, and BL9 have higher O/C ratios than other BLs (0.34–0.36). The aforementioned observation highlighted the impact of silica content and delignification degree on H/C and O/C ratios.

### Characterization of CNCs

**Fig. 1:**
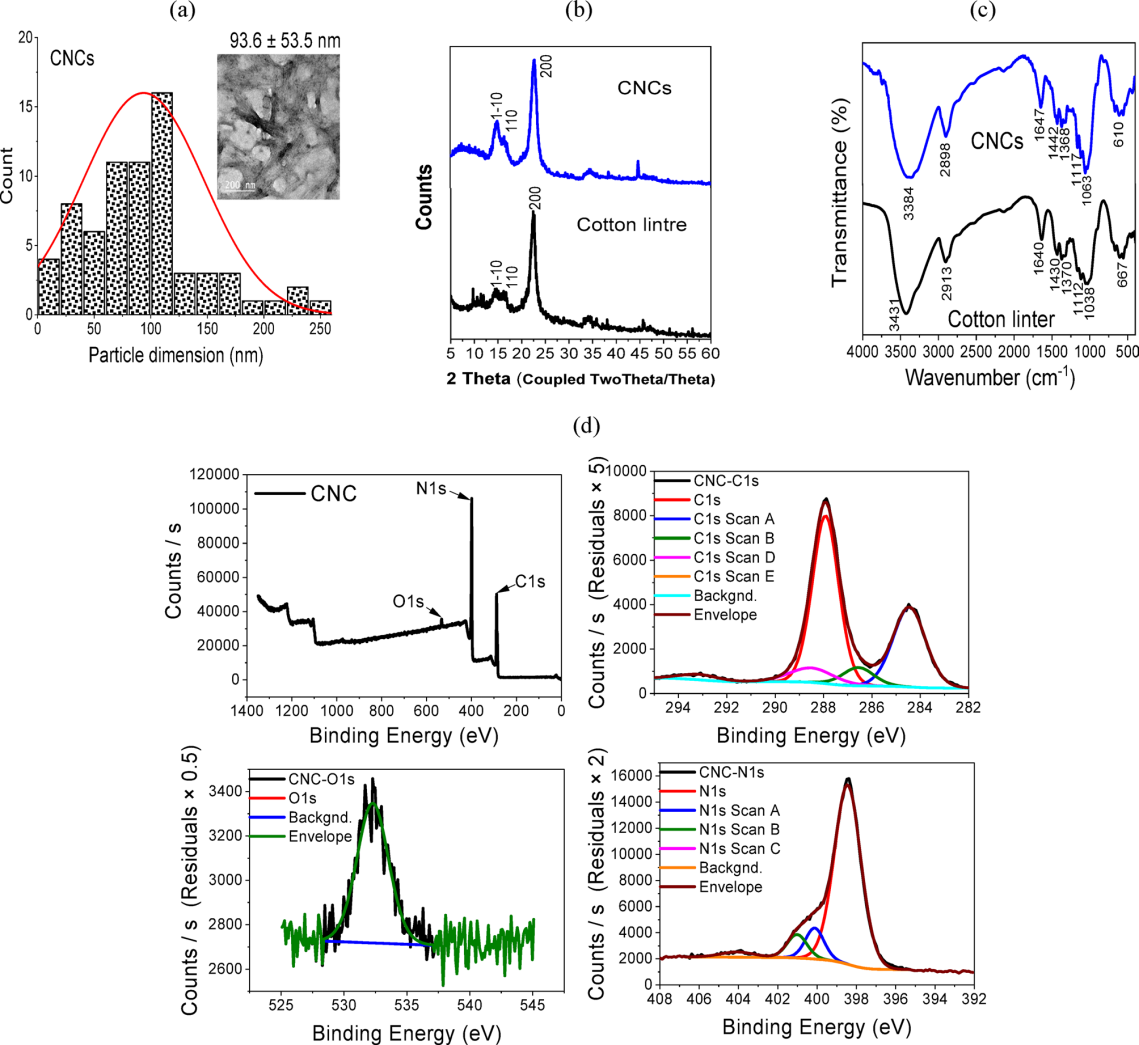
(**a**) Particle dimension distribution (PDD) & TEM images, (**b**) XRD, (**c**) FTIR and (**d**) XPS of CNCs.

The TEM graphs of the prepared CNCs are displayed in Fig. 1a to verify the preparation of particles in the nano range. The CNCs looked like rods or needles and were 93.6 ± 53.5 nm in length on average. There are less aggregated nanoparticles visible because anionic sulfuric ester groups repel one another. The dark spot in the TEM pictures was caused by phosphotungstic acid staining. The primary diffraction peaks in the XRD data of CNCs and native cotton linter are comparable and are located roughly at 2-theta values of 14.4, 15.13, 17.09, 20.83, and 22.84°, as shown in Fig. 1b. As a result, the diffraction planes (101), (10¯1), (021), and (002) are credited with these peaks, correspondingly^33^. The (040) plane is responsible for the weak peak area at 2theta = 34°, completing the CNCs pattern’s compatibility with mostly I type crystalline cellulose. The crystalline index (CI) of the produced CNCs was also estimated using XRD. The CI values of CNCs are substantially greater than those of native cotton linter. Furthermore, CNCs’ average crystallite size is 6.18 nm, which is less than cotton linter’s (8.11 nm) size.

Additionally, Fig. 1c shows the cotton linter’s ATR-FTIR spectra before and after hydrolysis. The stretching vibrations of -OH are responsible for the wide bands extending at 3384 to 3431 cm^− 1^, while the stretching vibrations of C–H are responsible for the bands near 2900 cm^− 1^. The distinctive bands at 1640, 1430, and 1370 cm^− 1^ are responsible for the in-plane bending vibrations of OH bending of adsorbed water, H-C-H, and O-C-H. Additional bands near 1115, 1050, and 660 cm^− 1^ are produced by the C-O-C stretching of the pyranose ring, the in-plane ring stretching vibration modes and the C-OH out of plane bending mode. In the OH band, the CNCs (cotton linter treated with H_2_SO_4_) show a red shift to a lower frequency, up to Δv 39 cm^− 1^. This is explained by the removal of the amorphous area from the cellulose structure.

XPS analysis was performed on the CNCs film employed in order to ascertain the basic composition of cellulose nanocrystals. Figure 1d shows three shoulder intensities at positions 288.3, 398.9, and 533.5 eV are seen in the XPS survey spectra of the CNCs film. These were identified as the carbon (C1s), nitrogen (N1s), and oxygen (O1s) distinctive peaks, respectively. Even following acid hydrolysis with H_2_SO_4_, the lack of a signal at 165 eV (S2p) indicates that sulfur is not present in the CNCs)^34^. The carbon of the C-C, C-O/C-OH/C-N, and O-C-O/C = O/C = N is shown by two peaks in the carbon high-resolution spectra at positions 284.6 eV and 288.3 eV. Two minor peaks, 400.0 eV and 401.1 eV, which represent C–N–C, N–(C)_3_, and N–H, are also visible, along with a major peak that is centered at 398.9 eV^35^. The shoulder peak that appeared at 533.5 eV is caused by the O-C-O/C-OH, based on the O1s high-resolution spectra. According to the results, the majority of the surface groups of CNCs were linked to carbon (47.5%) and oxygen (51.9%) as carboxyl and hydroxyl groups.

### Characterization of LNPs

#### Transmission electron microscope (TEM)

**Fig. 2:**
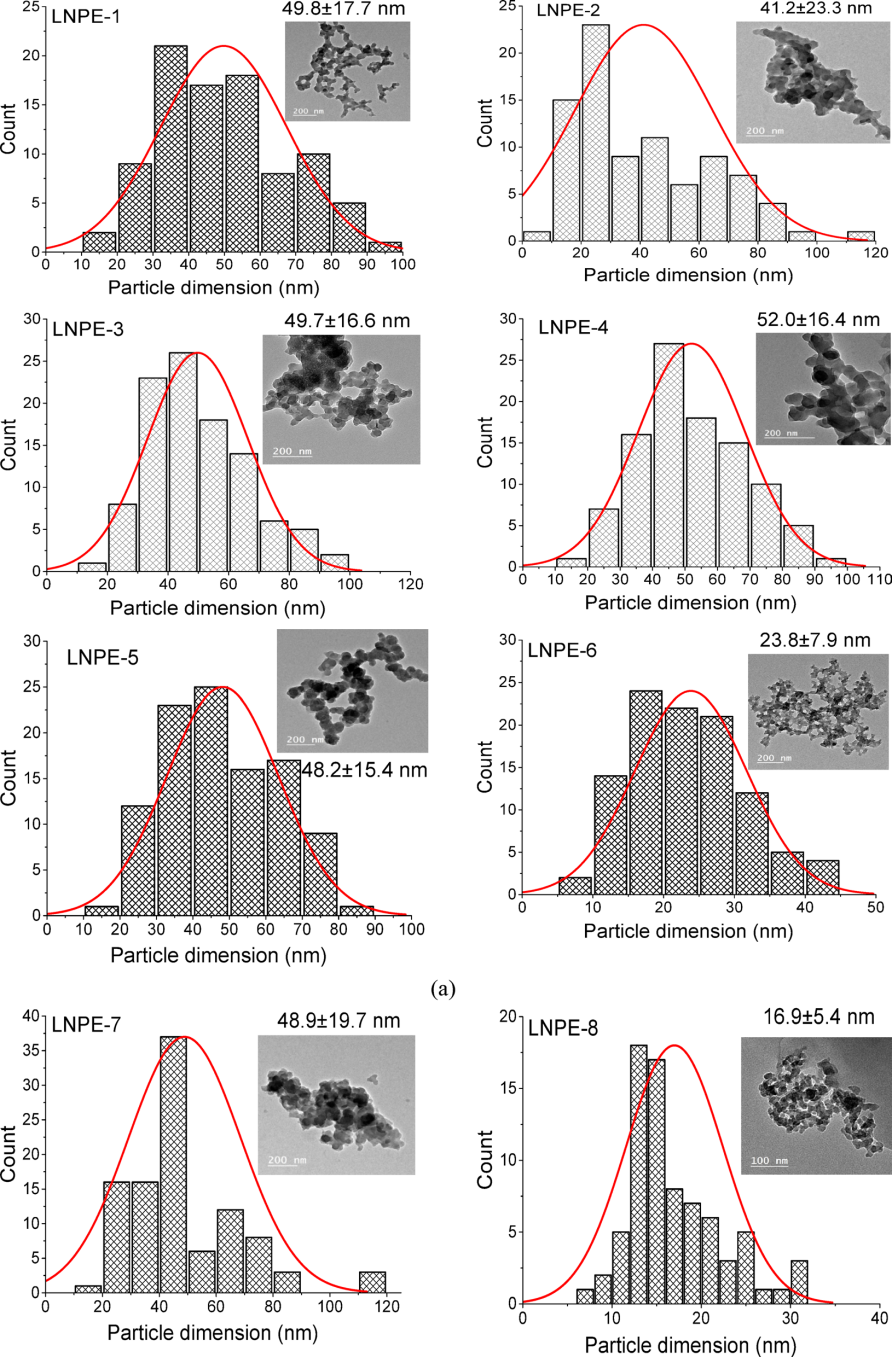

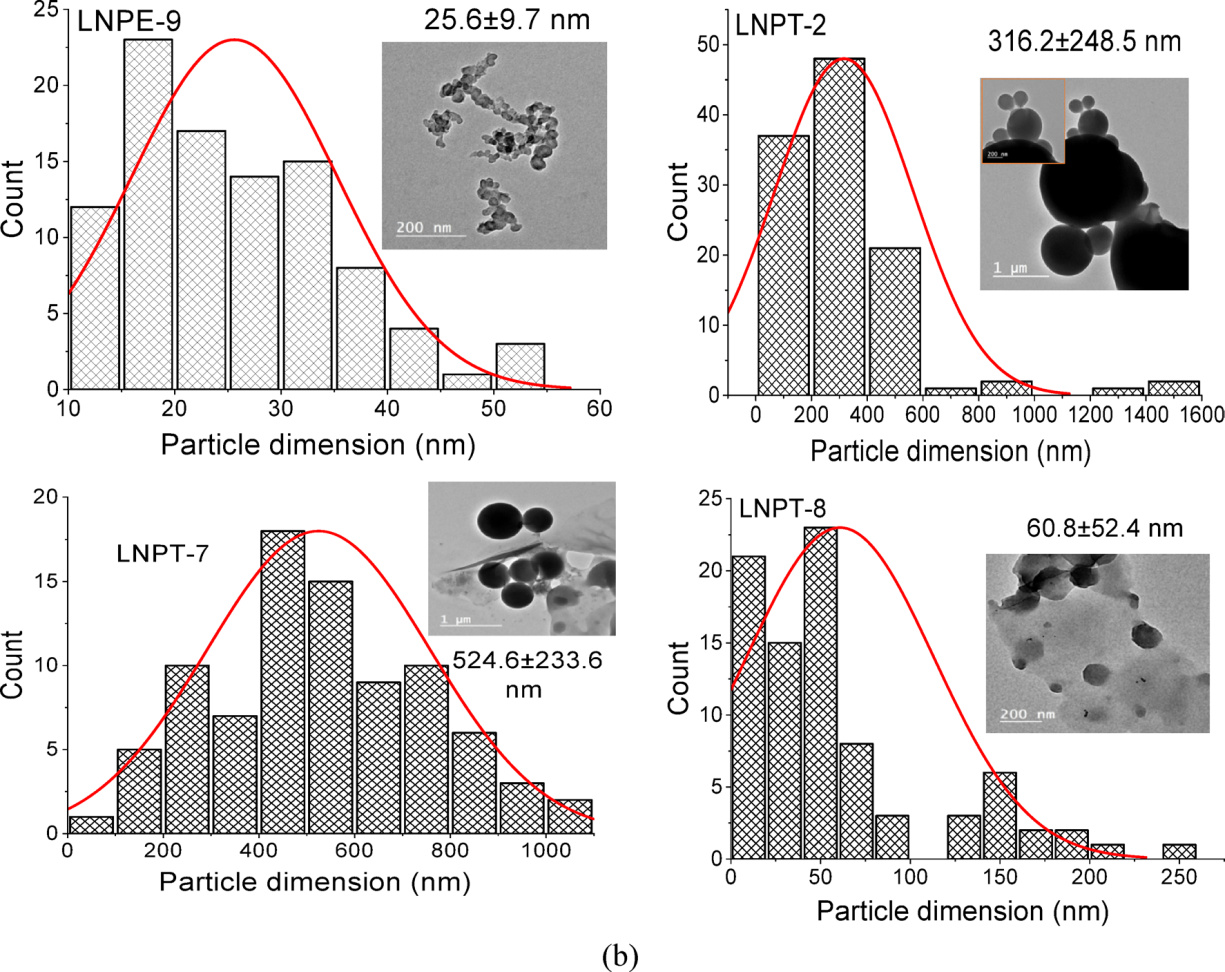
(**a**) Particle dimension distribution (PDD) and TEM images of lignin nanoparticles prepared from EG were coded LNPs (1-6). (**b**) Particle dimension distribution (PDD) and TEM images in comparison of LNPs produced from EG (LNPE 7–9) and THF LNPT (2, 7, 8).

The TEM photographs of the produced LNPs from various BLs precursors were displayed with their computed particle dimension distribution (PDD) in Fig. 2a and b. Table 2 includes the dimensions of the lignin nanoparticles as determined by ImageJ processing software (IJ 1.8) applying TEM photographs for 50–100 measurements. The developed nanoparticles appeared in a spherical or semi-spherical network structure in the TEM photographs. The average particle size of LNPs produced by employing THF (LNPT group) as a polar solvent is larger than that produced by utilizing EG (LNPE group). The mean diameters of LNPT and LNPE were 60.8 ± 52.4 to 524.6 ± 233.6 nm and 16.9 ± 5.4 to 52.0 ± 16.4 nm, respectively. This suggests that ethylene glycol, a polar solvent, performs well in obtaining LNPs with lower particle sizes as compared to tetrahydrofuran. Regarding the effectiveness of various pulping reagents on the distribution of dimensions, the findings demonstrated that alkaline soda pulping, with or without additives (AQ or BH) are present, had no discernible impact on the size of the resulting LNPs (LNPE1-LNPE5), whereas the particle size varied between 41.2 ± 23.3 and 52.0 ± 16.4 nm. Likewise, the LNPs derived from kraft pulping (LNPE7), which have an average particle size of 48.9 ± 19.7 nm, certainly show similar results for alkaline soda. The smallest LNP dimension (16.9 ± 5.4) was obtained by neutral-sulfite pulping with EG [LNPE8], which was followed by LNPs from alkaline-ammonia and sulfite pulping (23.8 ± 7.9 and 25.6 ± 9.7 nm, respectively).

**Table 2:** PDD of LNPs from different black liquors.

Reagent		PDD of LNPE, nm	PDD of LNPT, nm
NaOH	BL-1	49.8 ± 17.7	–
NaOH -AQ	BL-2	41.2 ± 23.3	–
NaOH-BH	BL-3	49.7 ± 16.6	–
NaOH-AQ-BH	BL-4	52.0 ± 16.4	–
M- NaOH -AQ-BH	BL-5	48.2 ± 15.4	316.2 ± 248.5
KOH-ammonia	BL-6	23.8 ± 7.9	–
Kraft	BL-7	48.9 ± 19.7	524.6 ± 233.6
Neutral-sulfite	BL-8	16.9 ± 5.4	60.8 ± 52.4
Acidic sulfite	BL-9	25.6 ± 9.7	

These data were compared with literature reports of LNP synthesis from other substrates and using other techniques. Nai et al., and Perera et al.^17,36^., reported TEM of LNPs extracted from alkali or Kraft pulping of empty fruit bunch (EFB) after ultrasonication or high shear homogenizer have mean particle size of LNPs at 220 and 283 nm. The average size of the nano-lignin particles produced by incubating Lactobacillus bulgaricus with rice straw is 101.6 nm after 24 h, 57.2 nm after 48 h, and 276.9 nm after 72 h^37^.

#### FTIR spectral analysis

**Fig. 3:**
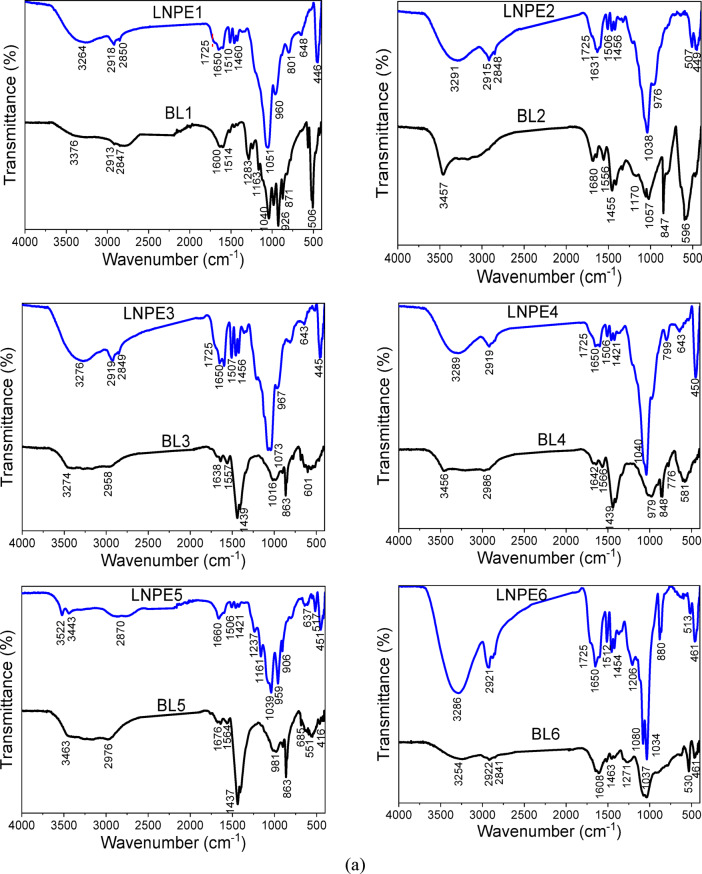

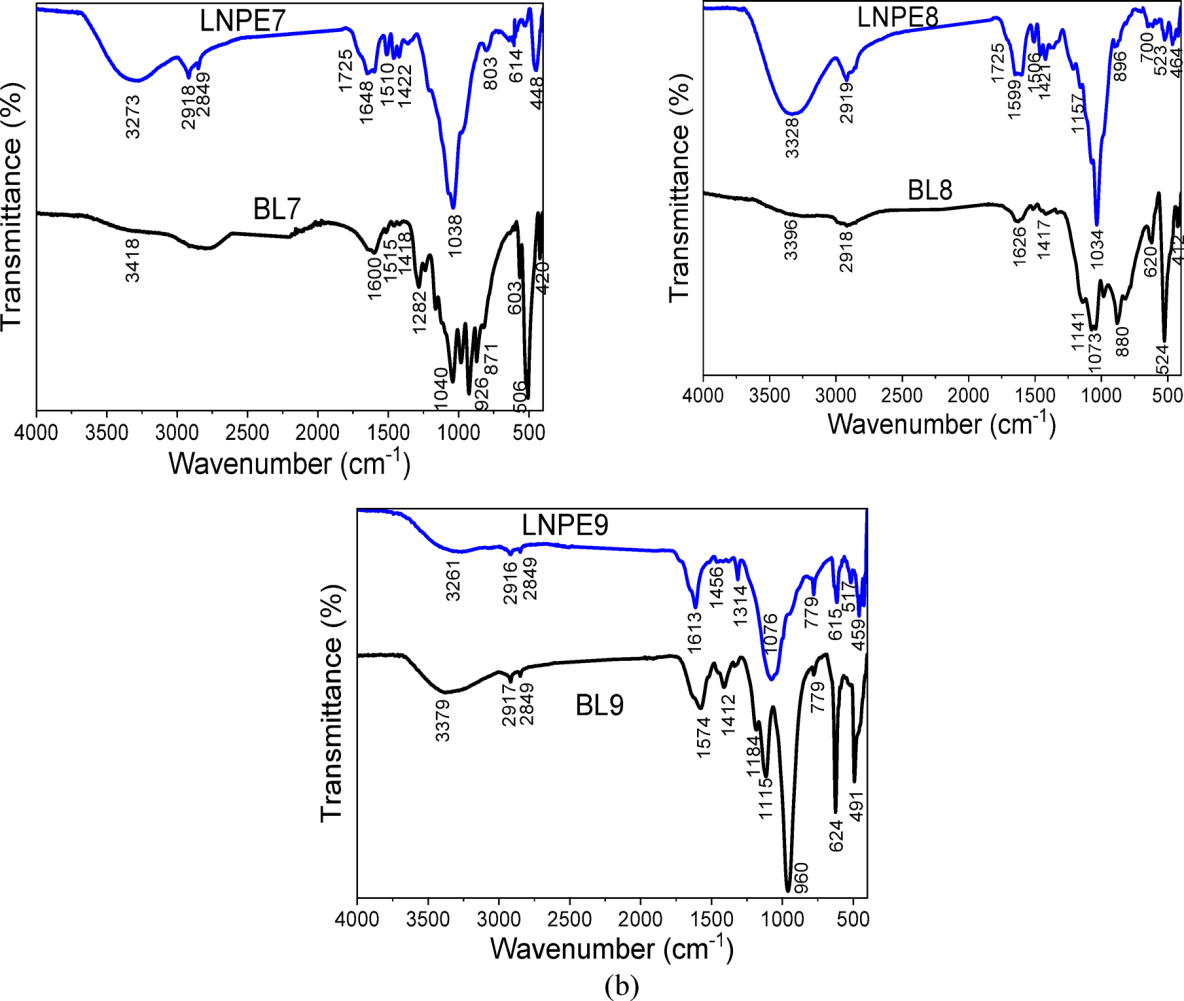
(**a**) FTIR of lignin nanoparticles prepared from EG (LNP1-LNPE6) versus their black liquors (BLs), (**b**) FTIR of lignin nanoparticles prepared from EG (LNPE7-LNPE9) versus their black liquors (BLs).

In comparison to their black liquors, Fig. 3a and b display the structural features and function groups of LNPE. All LNPE extracted using multiple approaches has FTIR spectra that are almost similar, with no discernible variation in the primary lignin characteristic peaks. Regarding BL, the lignin nanoparticles showed bands at 3286 cm^− 1^ (the hydroxyl group in lignin’s phenolic structures) and 2921 cm^− 1^ (C-H stretching in methylene and methoxy groups). The distinctive bands, which are about 1650, 1595, 1512, and 1454 cm^− 1^, are associated with the stretching vibrations of the unconjugated ketone, carboxylate group (COO), and aromatic skeleton (C = C, C-C, and C-H). At 1322 cm^− 1^, the syringyl unit exhibits C = O stretching, whereas the guaiacyl ring exhibits C–O stretching at 1206 cm^− 1^. The syringyl units exhibit C-O bending at 880 cm^− 1^ and C-H bending of the primary alcohols at 1034 cm^− 1^^30^.

There are some noticeable changes in the bands shift and intensity of several bands following the extraction of lignin nanoparticles from the BLs. After the synthesis of nano-lignin, a new shoulder is observed at 1725 cm^− 1^ that is related to C = O of LNPs. Beside a reduction in the OH stretching frequency was observed; this might be due to the loss of β-O-4 links as well as a decrease in hydrogen bonds within the lignin molecules. Additionally, the increased resonance of the oxygen function groups of the LNPs causes an increase in the band intensity of the OH stretching (at ~ 3200–3400 cm^− 1^) and C-O bending (at ~ 1034–1080 cm^− 1^). On the opposite side the band at 1454 cm^− 1^ (C = C) showed reduction in the band intensity due to the loss bond in the lignin structure.

### Evidence of CNC-LNP nanocomposites formation

#### ATR-FTIR analysis

**Fig. 4:**
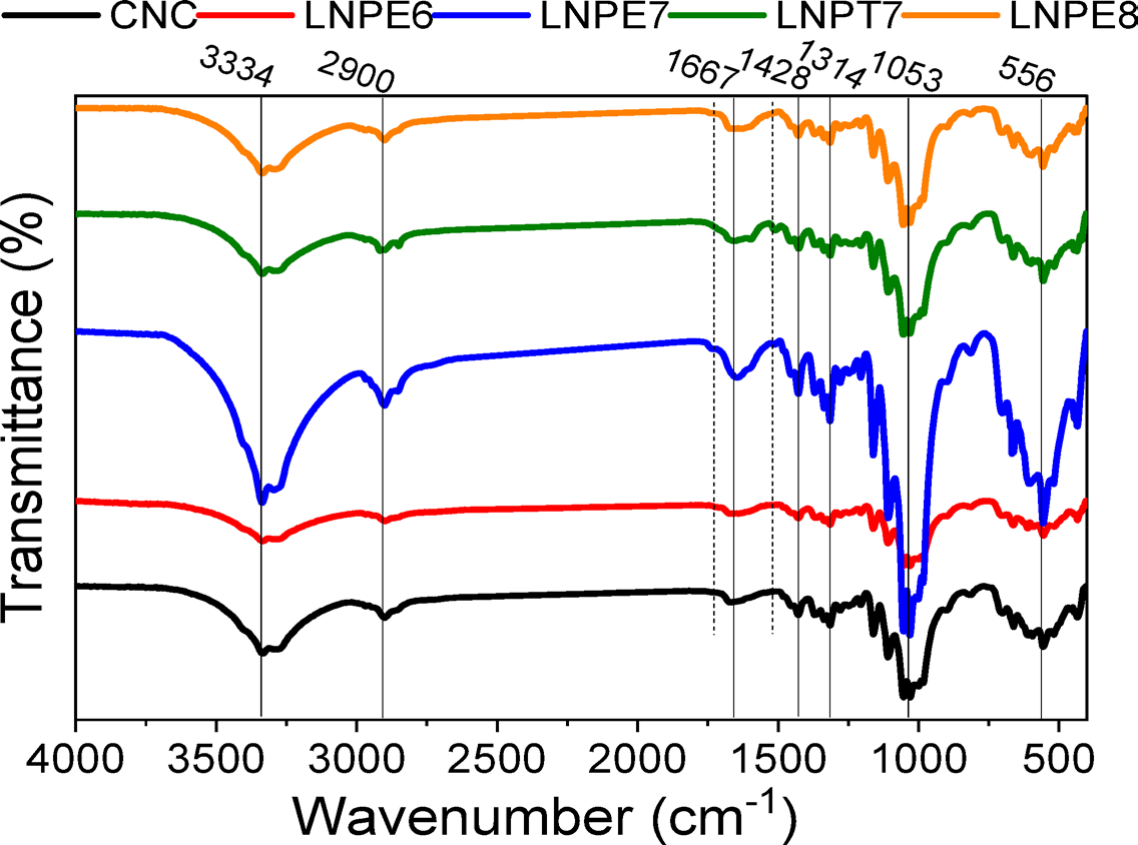
FTIR spectrum as a function of CNCs- based nanocomposites film containing selected LNPs.

The FTIR spectra of the spectra of the CNCs and CNC-LNP nanocomposites films are shown in Fig. 4. As previously mentioned^7,10^, the O–H stretching vibration group in the cellulose molecule is responsible for the peak at 3334 cm^− 1^ in the CNCs FTIR investigation. While OH bending vibrations of adsorbed water appeared up at 1667 cm^− 1^, cellulose C–H stretching groups displayed up at 2900 cm^− 1^. The H-C-H and O-C-H of cellulose appeared at 1428 and 1314 cm^− 1^. Additional bands near 1160, 1053, and 556 cm^− 1^ are assigned to the C-O-C stretching of the pyranose ring, the in-plane ring stretching vibration modes and the C-OH out of plane bending mode. This was demonstrated by the emergence of new shoulders at 1725–1735 cm^− 1^ and 1509–1515 cm^− 1^, which indicated the presence of some lignin function groups (C = O and C = C of the aromatic ring of lignin, respectively). A possible explanation for the shift in some bands’ intensities is the creation of hydrogen bonds between the hydroxyl groups of CNCs and the OH of LNPs. These findings show that the isolated LNPs has been incorporated into the cellulose nanoparticles in the nanocomposite.

#### XRD test

**Fig. 5:**
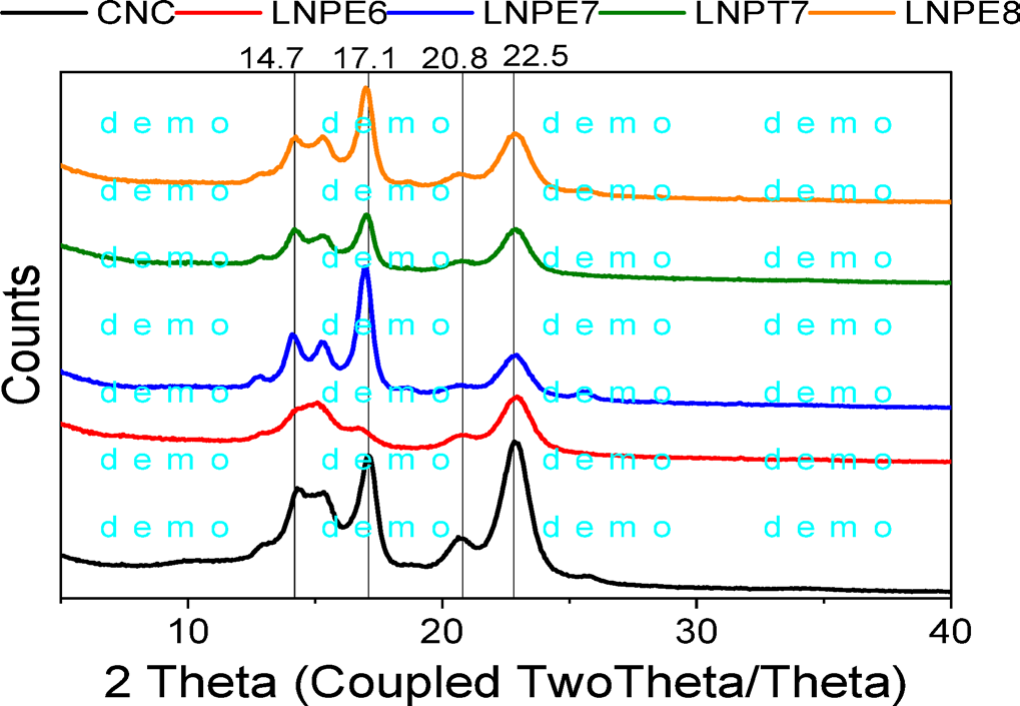
XRD spectrum as a function of CNCs- based nanocomposites containing selected LNPs.

Figure 5 displays the XRD patterns of the CNCs and modified CNC-LNP nanocomposite film. The figure illustrates that the XRD pattern of CNCs displayed the distinctive cellulose I peaks in accordance with previous studies^38^. A large peak at 2θ = 22.5°, two overlapping smaller peaks at 2θ = 14.7° and 16.7°, and a weak peak at 2θ = 17.09, 20.83° were all present in the XRD pattern of CNCs. The planes (002), (101), (10¯1) and (021) are represented by the diffraction peaks, respectively. Table 3 displays several XRD properties, including the crystalline index (CI) and crystallite size. It has been noted that CNC-LNP nanocomposites have a damaged crystalline structure. For pure CNCs, the crystallinity index (CI) drops from 68.19 to 57.77, 54.32, 57.93, and 57.98% for CNC-LNPE6, CNC-LNPE7, CNC-LNPT7, and CNC-LNPE9, respectively. This suggests that the lignin nanoparticles may have contributed to the reduction in CNCs’ CI by destroying the original crystalline structure. This was identified as the result of the modified CNCs possibly hindering the CNCs’ ability to recrystallize by decreasing chain mobility^38^. Using the Scherrer formula, the crystallite size corresponding to the (200) plane of CNC (10.03 nm) was marginally lower than that of CNC-LNP nanocomposites (10.1–11.54 nm). The crystallites of CNC-LNP nanocomposites in the (110) plane is greater in size ranging from 83.66 to 83.75 nm, whereas those of CNCs are 80.5 nm.

**Table 3:** PDD of LNPs from different black liquors.

Sample code	Pos. (2Ɵ degree)	d-spacing (A^o^)	FWHM (degree)	Crystallite size (nm)	D (nm)	Crystallinity (%)
CNC	14.4	6.425	0.1	83.67	56.05	68.19
	15.13	5.882	0.104	80.51		
	17.09	5.232	0.388	21.63		
	20.83	4.291	0.1	84.4		
	22.84	3.898	0.844	10.03		
CNC-LNPE6	14.34	6.17	0.1	83.66	65.47	57.77
	15.09	5.866	0.1	83.74		
	20.75	4.277	0.1	84.39		
	22.94	3.874	0.839	10.1		
CNC-LNPE7	12.85	6.88	0.1	83.53	51.27	54.32
	14.18	6.24	0.1	83.65		
	15.25	5.80	0.1	83.75		
	16.96	5.22	0.391	21.46		
	20.78	4.27	0.1	84.39		
	22.91	3.88	0.774	10.94		
	25.56	3.48	0.363	23.45		
CNC-LNPT7	12.91	6.85	0.1	83.54	70.91	57.93
	14.27	6.20	0.1	83.65		
	15.25	5.80	0.1	83.75		
	17.01	5.21	0.437	19.21		
	20.76	4.28	0.1	84.39		
	22.88	3.88	0.734	11.54		
CNC-LNPE8	12.86	6.88	0.1	83.53	70.91	57.98
	14.32	6.18	0.1	83.66		
	15.30	5.79	0.1	83.75		
	16.99	5.21	0.411	20.42		
	20.74	4.28	0.1	84.38		
	22.85	3.89	0.795	10.65		
	25.59	3.48	0.542	15.7		

#### Atomic force microscope (AFM)

**Fig. 6:**
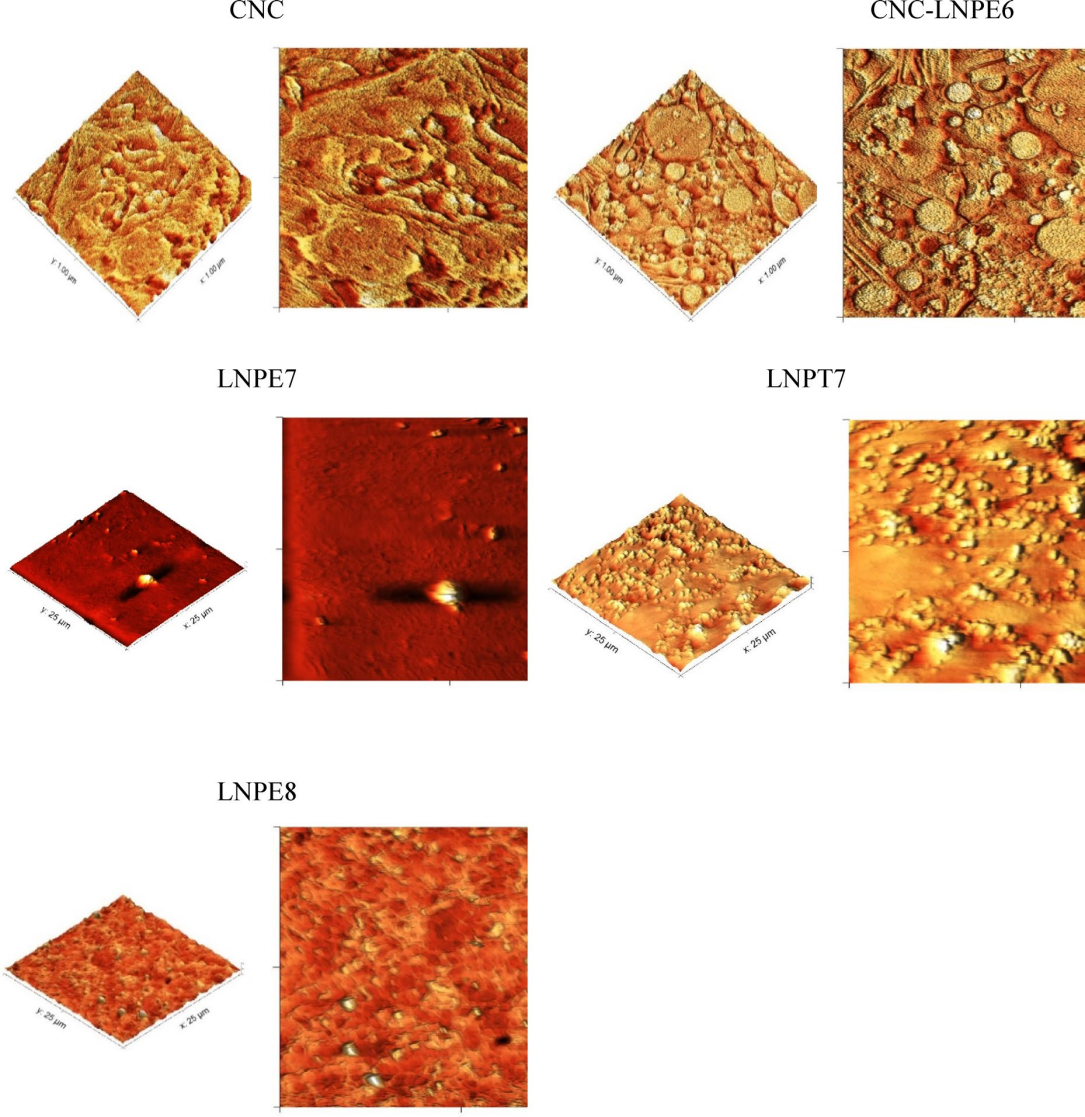
AFM topography images of CNCs- based nanocomposites containing selected LNPs.

The surface characteristics of the CNCs and CNC-LNPs nanocomposites on glass substrates were examined using AFM. The topographic pictures of the selected films are displayed in Fig. 6.

The CNCs film surface’s three-dimensional morphology shows rough, consistent structural elements with some connected needle shapes. As evidenced by the 2D and 3D morphologies of spherical or dot particles with sizes ranging from 0.11 to 0.15 μm, the LNP nanoparticles evenly dispersed in the CNC polymer matrix. There were no discernible agglomerations or clusters over the whole broken surface, suggesting that the modified CNC films had a compact, dense, and CNC-LNP provided by topography AFM pictures. LNPs are included into the resulting modified CNC film surface, which may be characterized as a less rough nanocomposite film. Mean roughness measurements of the modified CNC are as follows: 14.53 nm for CNC-LNPE6, 15.22 nm for CNC-LNPE7, 39.67 nm for CNC-LNPT7, and 12.32 nm for CNC-LNPE8. The CNC films have an average roughness of 250.9 nm.

#### Polarizing optical microscopy (POM)

**Fig. 7:**
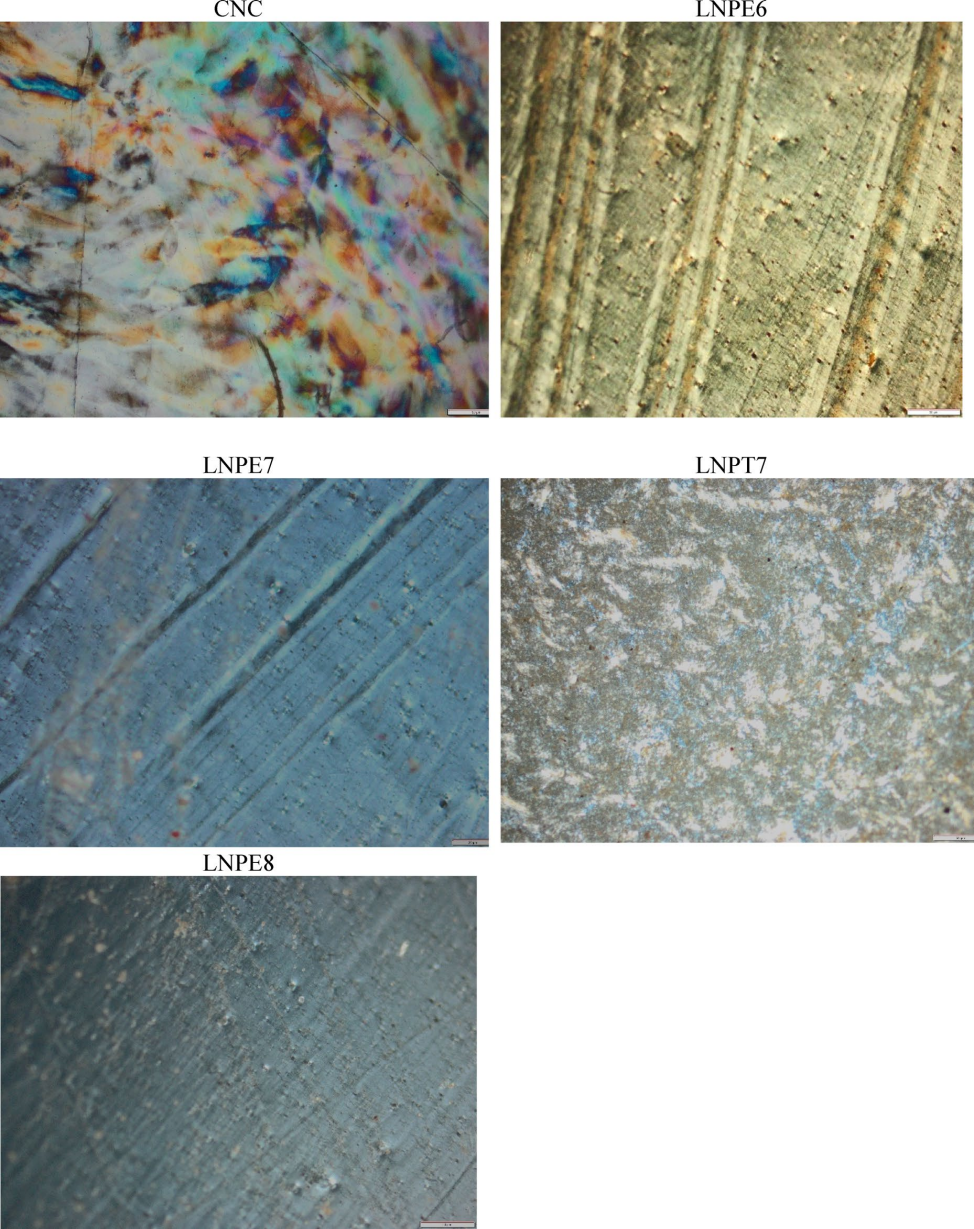
AFM topography images of CNCs- based nanocomposites containing selected LNPs.

The transmitted cross-polarized optical microscopy images (POM) of the chosen CNC-LNPs nanocomposites and the CNC films are displayed in Fig. 7. As a result, CNC rods self-assemble, the anisotropic phase of CNCs across the polarizer exhibits a structure that is helical and a colorful pattern, like a fingerprint^39^. For CNCs-LNPE6, LNPE7, and LNPE8, this chiral nematic (colorful) texture totally changes into a yellow, blue or black with aligned vertically optical picture. The CNC-LNPT7, on the other hand, displayed an arrangement of CNCs with the spherical large particle in a straightforward anisotropic phase that maintained its birefringence and had a uniform interference color. Based on their remarkable polarizing properties, the nanocomposites are more useful and a feasible biobased film material for applications that need visible light polarization and UV protection, such as contact lenses, sunglasses, windows, etc.

#### UV blocking property

**Fig. 8:**
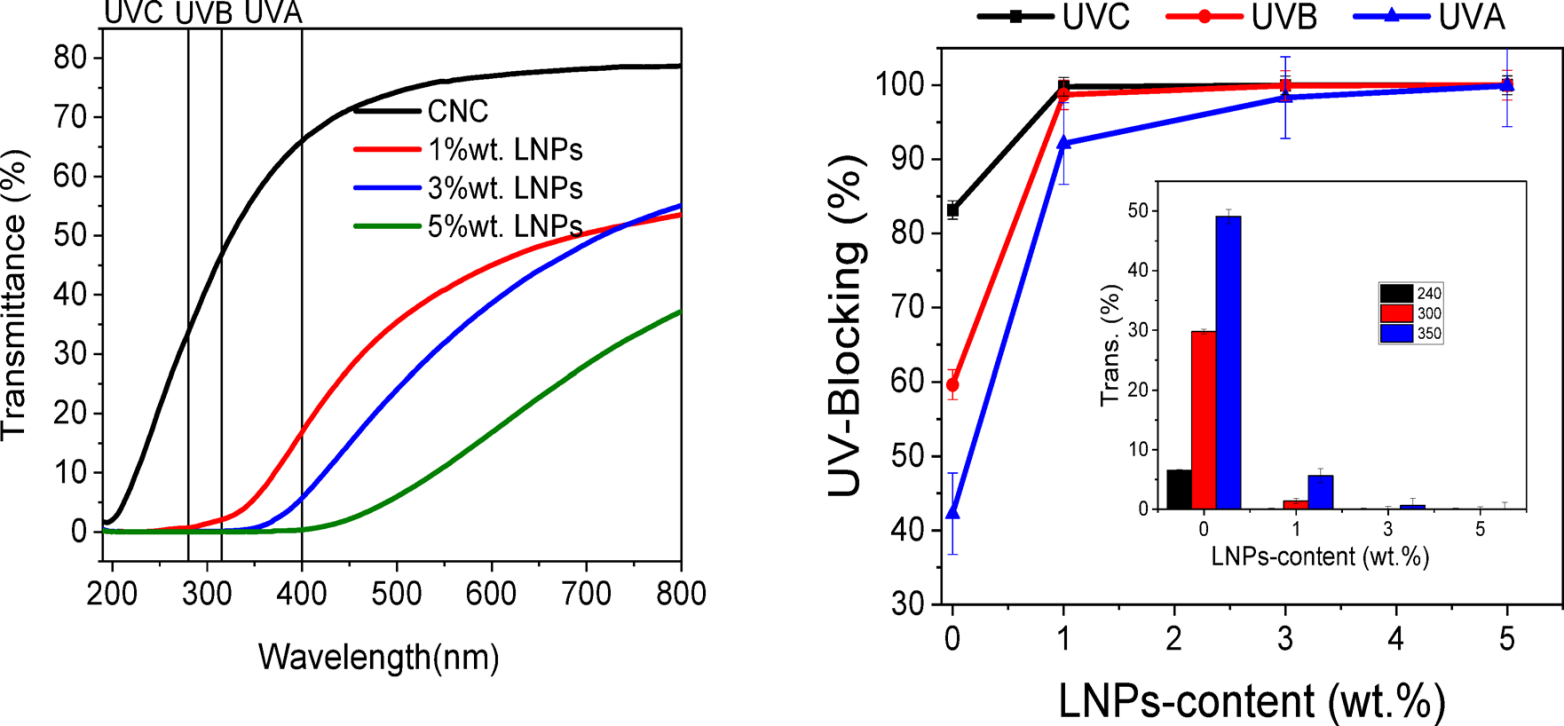
Optical UV–Vis. transmittance spectrum as a function of the wavelength of CNC-based nanocomposites containing different ration of LNPs.

The transmittance of the suspension of CNC-LNP -nanocomposites with varying ratios of LNPE6 within 190 to 800 nm UV–Vis. light range and the UV light-blocking at three different stages (UVC, UVB, and UVA) are shown in Fig. 8. The UV blocking of CNC suspension at UVC, UVB, and UVA stages was 83.2, 59.6, and 42.2%, respectively. LNPs, as anticipated, lower the transmittance of the CNCs suspension; interestingly, the spectra profile changes according to the nanoparticle content. Gradually increasing the nanoparticle content from 1 to 5 weight% reduces transmittance and increases UV-blocking. Using LNPs also makes the suspension less transparent. The transparency of nanocomposites to UV-visible light wavelengths is reduced due to the high color level of lignin^40^. At about 315 nm, a significant decrease in transmittance was seen in all nanocomposite solutions containing LNPs, suggesting an effective protection against UVB and UVC rays (98.7–99.9%).

The CNC-LNP nanocomposites including LNPE6 had a higher shielding effect, particularly when 3 and 5 wt % of LNPs were included. They demonstrated total blocking of the UV area (98.3–99.9%) and limited transmission of the visible region. It’s significant to note that UV blocking at the UVA region may reach 92.1% with a relatively low LNPs concentration of 1 wt %. One possible explanation for LNPs’ capability to block UV light is the presence of UV chromosphere functional groups, such as quinones and methoxy substituted phenoxy groups that can be conjugated with double bonds or carbonyl functional groups^41^. The following is an illustration of the suggested mechanism of LNPs as a UV protectant. When LNPs are exposed to ultraviolet light, electrons migrate and a carbonyl group is formed, which serves to block the UV rays. Through the conversion of photon energy into heat energy or re-emission of photon energy at a lower energy, CNC-LNPs nanocomposite suspension efficiently suppresses UV rays. LNPs have a remarkable ability to absorb ultraviolet light due to their complex structure, which is composed of aromatic rings and a variety of functional and chromophore groups joined by ether or single bonds^41^.

**Fig. 9:**
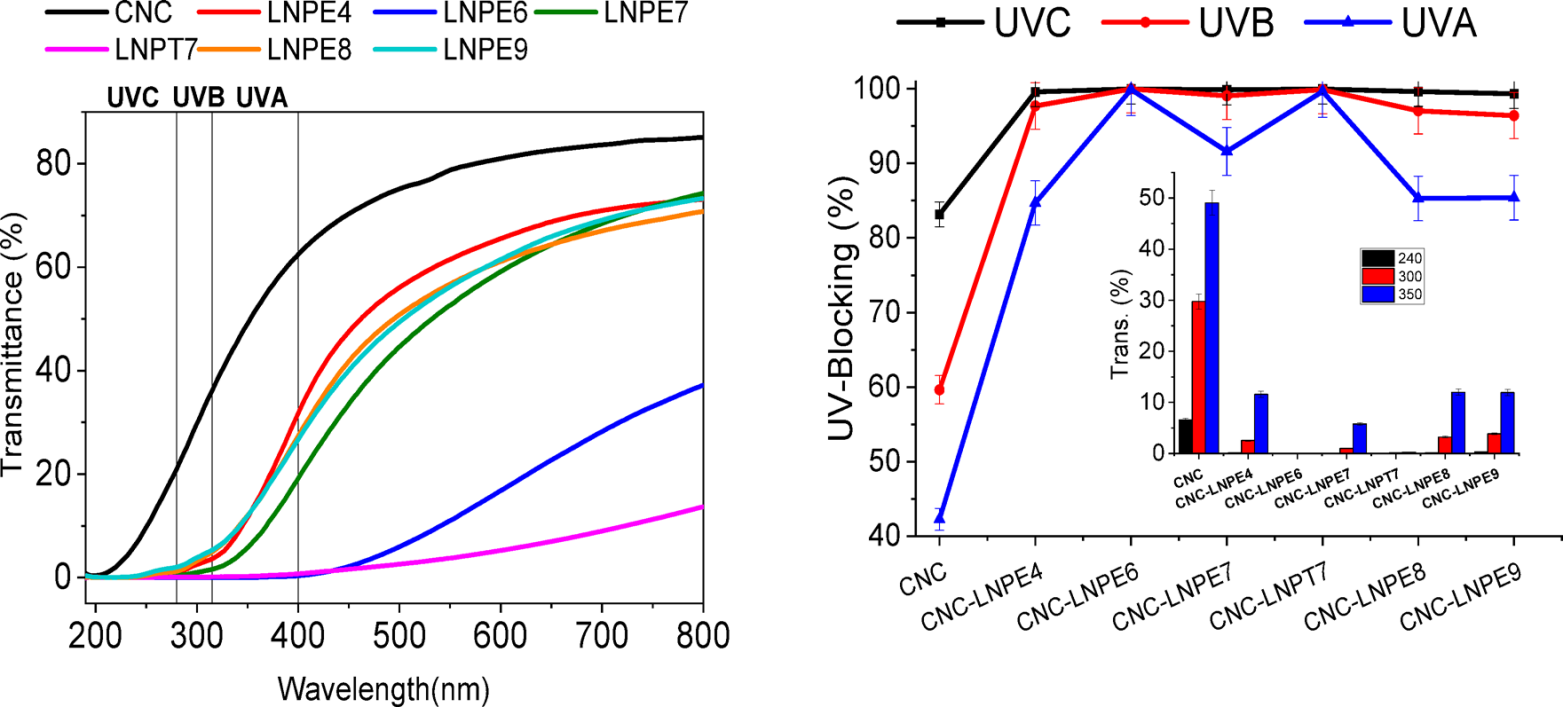
Optical UV–Vis. transmittance spectrum as a function of the wavelength of CNC-based nanocomposites containing different LNPs.

In an effort to evaluate the role of pulping reagents on the preparation of LNPs as well as the dimension of the resulting LNPs affect the UV blocking properties of CNC-LNP nanocomposites, several LNP samples derived from BLs of different pulping agents—NaOH-AQ-BH, KOH/NH_4_OH, Kraft, neutral, and acidic sulfite—are selected for additional analysis. The optical UV-Vis transmittance spectrum of CNC-LNP nanocomposites with 5% of the different LNPs as a function of wavelength is displayed in Fig. 9. The behavior of selected LNPs on UVA blocking of CNCs is significantly varied according to type of pulping agents. The results showed that LNPs produced from Kraft (Via THF; LNPT7) and KOH/NH_4_OH (LNPE6) with the highest shielding effect, followed by Kraft (Via EG; LNPE7). In contrast, CNCs-LNPE7 achieved 91.6% UV-blocking, whilst nanocomposites based on LNPE6 and LNPT7 had 99.9 and 99.7% UV-blocking, respectively. The shielding effect of the remaining samples, LNPE4, LNPE8, and LNPE9, was lower than the others and was almost the same (84.7–85.4%). The LNPT7 characterized with the largest complete spherical particles 524.6 ± 233.6 nm, while LNPE6 characterized with semi-spherical particle of moderate size 23.8 ± 7.9 nm. The findings demonstrated no correlation between the particle dimension distributions of the LNPs being studied and the UV blocking of CNC-LNP nanocomposites.

In comparison to other UV shielding systems that employed a variety of UV blocking agents^42–48^, including modified CNF, nano-lignin, nano-silver, carbon quantum dot, tannic acid, cinnamonaldehyde, and nano zinc oxide, as well as polymer matrices like chitosan, nanocellulose, micro-fibrillated cellulose, polyvinyl alcohol and starch, this study’s use of cellulose nanocrystal/LNPs hybrid nanocomposites demonstrated an impressive UV blocking efficiency of about 100% (Table 4). Notably, the resulting solution outperformed those containing curcumin grafted CNF, lignin/silver nanoparticles, tannic acid, and cinnamon aldehyde.

**Table 4:** Comparison of UV blocking efficiency of different agents.

UV Blocking agent	Polymer	UV-Blocking efficiency (%)	Reference
Curcumin cellulose nanofibers (10%)	Chitosan	77.4%	^42^
Lignin-silver nanoparticles (Lig-Ag NPs) (10–15%)	Micron-sized cellulose fibers (CMF) /cellulose nanofibers (CNF) film	About 85-98.2- UVA (%)	^43^
Cinnamaldehyde	Corn starch	30%	^44^
Sheet or belt -like ZnO nanostructures (1–6%)	Nanocellulose	About 90%	^45^
Halloysite nanotubes-zinc oxide (1–20%)	Cellulose nanofibrils	95.7 UVA (%)	^46^
Carbon dot (40 wt %)	Polyvinyl alcohol (PVA)	> 90%	^47^
Tannic acid (5–20%)	Micro-fibrillated cellulose	55.8–85% UVA 98% UVB	^48^
LNPE6 (5 wt%)	Cellulose nanocrystal	99.9 ~ 100% UVA	Present study
LNPT7 (5 wt%)	Cellulose nanocrystal	99.6 ~ 100% UVA	Present study

## Conclusions

This work deals with Selecting the pulping black liquor as precursor of nano-Lignin in the development of nanocrystalline cellulose-composite for UV‑Light Blocking, as an environmentally friendly and economical route. The impact of changing the pulping black liquor of the resulting LNPs, as a result of changing the pulping reagent, was examined. In the production process, preliminary study was carried out on one black liquor to optimize the percentage of LNP blended with CNC that provided strong UV-blocking properties. Further study was performed on LNPs from other BLs to determine which was the best. Along with the various methods evaluated to demonstrate the production of CNC-LNP nanocomposites, the results showed that the various LNPs had a good barrier against UVB and UVC rays (96.4–99.9%). While, the behavior of LNPs on UVA blocking of CNCs varies greatly depending on the kind of pulping agent. The nanocomposites based on lignin nanoparticles (LNPE6) extracted from BL of KOH-NH4OH and kraft reagent (LNPT7), using ethylene glycol and tetrahydrofuran solvents exhibited the highest shielding effect, with 99.9 and 99.7%, respectively. Additionally, these results are superior to shielding materials described in the literature, such as curcumin-grafted CNF, lignin/silver nanoparticles, tannic acid, and cinnamonaldehyde. The findings demonstrate that the developed environmentally friendly nanocomposites from undesirable wastes (Rice straw and its pulping black liquor) supports sustainable material innovation and offers plenty of potential for UV protection applications, such as active packaging and eyewear.

## Data Availability

All data are included the article.
